# Novel truncation mutations in *MYRF* cause autosomal dominant high hyperopia mapped to 11p12–q13.3

**DOI:** 10.1007/s00439-019-02039-z

**Published:** 2019-06-06

**Authors:** Xueshan Xiao, Wenmin Sun, Jiamin Ouyang, Shiqiang Li, Xiaoyun Jia, Zhiqun Tan, J. Fielding Hejtmancik, Qingjiong Zhang

**Affiliations:** 1 State Key Laboratory of Ophthalmology, Zhongshan Ophthalmic Center, Sun Yat-sen University, 54 Xianlie Road, Guangzhou, 510060 China; 2 Institute for Memory Impairments and Neurological Disorders, University of California, Irvine, CA USA; 3 Ophthalmic Genetics and Visual Function Branch, National Eye Institute, National Institutes of Health, Bethesda, MD USA

## Abstract

High hyperopia is a common and severe form of refractive error. Genetic factors play important roles in the development of high hyperopia but the exact gene responsible for this condition is mostly unknown. We identified a large Chinese family with autosomal dominant high hyperopia. A genome-wide linkage scan mapped the high hyperopia to chromosome 11p12–q13.3, with maximum log of the odds scores of 4.68 at theta = 0 for D11S987. Parallel whole-exome sequencing detected a novel c.3377delG (p.Gly1126Valfs*31) heterozygous mutation in the *MYRF* gene within the linkage interval. Whole-exome sequencing in other 121 probands with high hyperopia identified additional novel mutations in *MYRF* within two other families: a de novo c.3274_3275delAG (p.Leu1093Profs*22) heterozygous mutation and a c.3194+2T>C heterozygous mutation. All three mutations are located in the C-terminal region of MYRF and are predicted to result in truncation of that portion. Two patients from two of the three families developed angle-closure glaucoma. These three mutations were present in neither the ExAC database nor our in-house whole-exome sequencing data from 3280 individuals. No other truncation mutations in *MYRF* were detected in the 3280 individuals. Knockdown of *myrf* resulted in small eye size in zebrafish. These evidence all support that truncation mutations in the C-terminal region of *MYRF* are responsible for autosomal dominant high hyperopia in these families. Our results may provide useful clues for further understanding the functional role of the C-terminal region of this critical myelin regulatory factor, as well as the molecular pathogenesis of high hyperopia and its associated angle-closure glaucoma.

**Electronic supplementary material:**

The online version of this article (10.1007/s00439-019-02039-z) contains supplementary material, which is available to authorized users.

## Introduction

High hyperopia, defined as cycloplegic sphere refraction ≥ + 5.00 diopters (D) (Association 2006) or ocular axial length < 21 mm (Carricondo et al. 2018; Fuchs et al. 2005), is a common severe refractive error. High hyperopia is present in approximately 0.8–3.0% of children between 3 and 6 years old, according to population studies (Giordano et al. 2009; Multi-Ethnic Pediatric Eye Disease Study 2010). This condition is frequently associated with blurred vision, asthenopia, accommodative and binocular dysfunction, amblyopia, strabismus, or even primary angle-closure glaucoma (Association 2006; Jonas et al. 2017; Klimek et al. 2004; Shen et al. 2016). Most cases with high hyperopia are characterized by a normal corneal diameter and a short axial length, with no other significant ocular or systemic anomalies. High hyperopia is also observed in other ocular or systemic diseases, such as microphthalmia (Gal et al. 2011; Sundin et al. 2005), Leber congenital amaurosis (Abouzeid et al. 2006), retinal dystrophy (Bifari et al. 2016), and Alstrom syndrome (Khan et al. 2015), in which microphthalmia accompanies a small cornea, while others have retinal degeneration.

Two other extreme forms of high hyperopia have been frequently described: posterior microphthalmia and nanophthalmos. Both of these conditions feature axial length < 20 mm and extremely high hyperopia (usually ≥ + 8 D) (Altintas et al. 1997; Nowilaty et al. 2013a; Park et al. 2016; Vingolo et al. 1994). Posterior microphthalmia usually features a normal-appearing anterior segment but is frequently associated with typical papillomacular fold, sclerochoroidal thickening, and/or several other related changes to the posterior fundus (Erdol et al. 2008; Goldblum and Mojon 1999; Khairallah et al. 2002; Nowilaty et al. 2013b; Park et al. 2016; Spitznas et al. 1983). Nanophthalmos is characterized by a structurally normal but generally small eye, with the anterior and posterior segments both being affected. Apart from microcornea and a shallow anterior chamber, nanophthalmos may also feature fundus changes similar to those observed in posterior microphthalmia (Helvacioglu et al. 2014; Yalcindag et al. 2011). Biometric and molecular characterization suggests that posterior microphthalmia and nanophthalmos both belong to a spectrum of high hyperopia but are not distinct phenotypes (Nowilaty et al. 2013a). This characterization is further supported by mutations in *MFRP* in different families with nanophthalmos (Sundin et al. 2005), high hyperopia (Xu et al. 2016), or posterior microphthalmia (Aldahmesh et al. 2011; Matsushita et al. 2012; Wasmann et al. 2014). Similarly, mutations in *PRSS56* (Gal et al. 2011; Nair et al. 2011; Orr et al. 2011; Said et al. 2013) have been identified in patients with either posterior microphthalmia or nanophthalmos.

Genetic factors play important roles in the development of high hyperopia. In the clinic, high hyperopia may demonstrate an autosomal dominant or autosomal recessive pattern of inheritance in families, although most cases are sporadic. Families with high hyperopia have been reported previously (Fledelius et al. 2004; Fuchs et al. 2005). Family aggregation studies, twin studies, and genome-wide association studies all support a genetic contribution to hyperopia (Dirani et al. 2006; Hammond et al. 2001; Lee et al. 2001; Simpson et al. 2014; Wojciechowski et al. 2005). Four loci for nanophthalmos, i.e., NNO1 for autosomal dominant (Othman et al. 1998), NNO2 for autosomal recessive (Sundin et al. 2005), NNO3 for autosomal dominant (Li et al. 2008), and NNO4 for autosomal dominant (Awadalla et al. 2014), have been mapped, in which mutations in *MFRP* are responsible for NNO2 while those in *TMEM98* are responsible for NNO4. Apart from *MFRP* and *TMEM98*, high hyperopia is a frequent sign in other ocular or systemic diseases associated with mutations in several following genes, including *PRSS56* (Gal et al. 2011), *CRB1* (Abouzeid et al. 2006), *LCA5* (den Hollander et al. 2007), *IFT140* (Bifari et al. 2016), and *KERA* (Khan et al. 2004). However, most patients with high hyperopia do not have mutations in these genes, according to our in-house unpublished data and our previous studies (Jiang et al. 2013; Wang et al. 2009; Xu et al. 2016).

In the current study, genome-wide linkage analysis mapped high hyperopia in a large family to chromosome 11p12–q13.3. Whole-exome sequencing identified three novel truncation mutations in *MYRF* located in the linkage interval in three families with high hyperopia, including the family mapped to this locus. Sanger sequencing confirmed the mutations and their cosegregation with high hyperopia in the three families. All patients with high hyperopia were free of other systemic anomalies, including cardiac and urogenital anomalies. Two patients from two families developed angle-closure glaucoma. Additional analyses of mutation prediction, mutations in the database, mutation location, and *myrf* knockdown in zebrafish all support that truncation mutations in the C-terminal region of this critical transcriptional regulator for central nervous system myelination are responsible for high hyperopia that is associated with angle-closure glaucoma.

## Materials and methods

### Families and clinical data

**Fig. 1:**
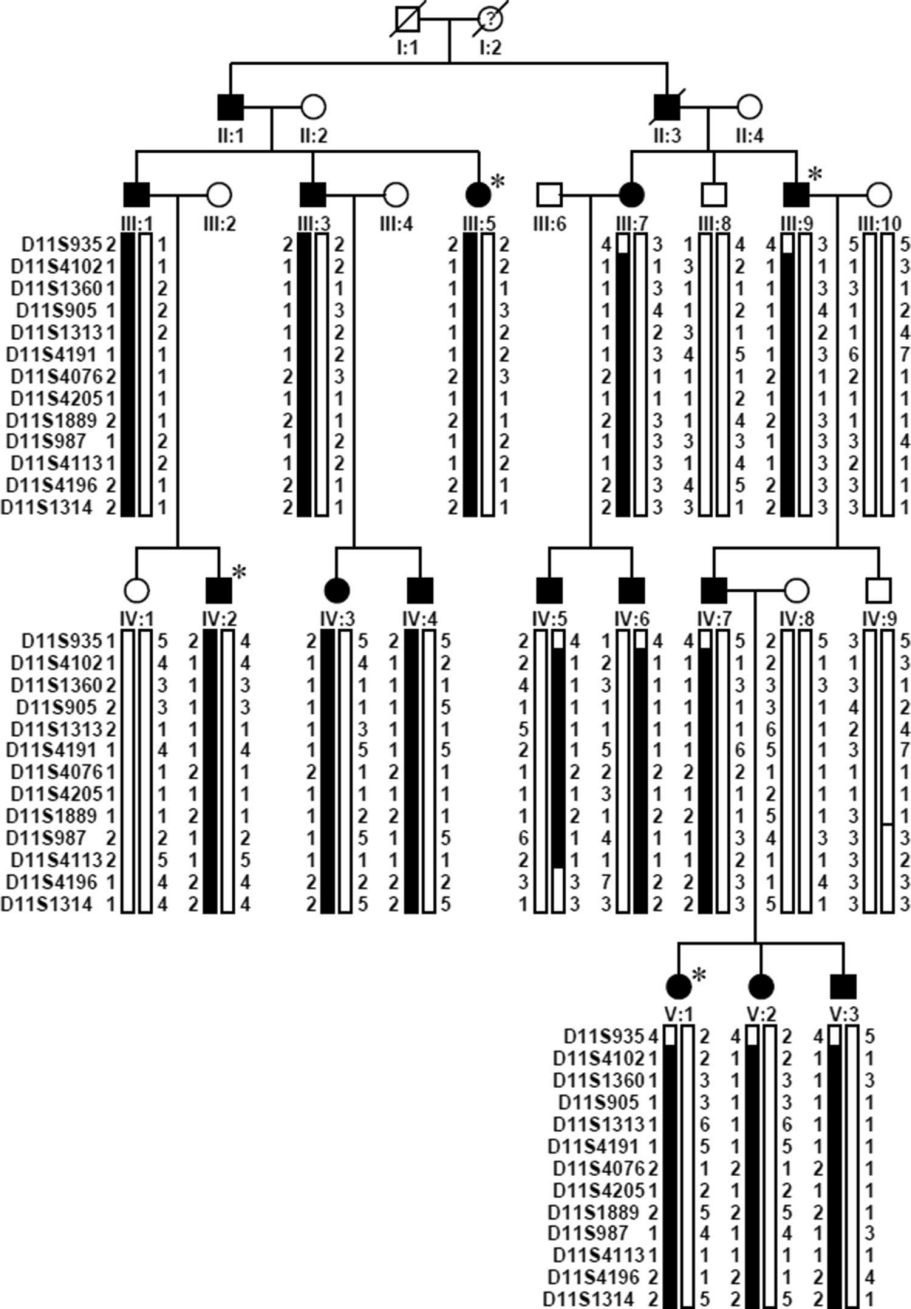
Pedigree and haplotype diagram of family ZOC710536. Filled squares/circles represent male/female patients with high hyperopia, while other squares/circles represent unaffected male/female individuals. Black bars indicate the haplotype with the disease allele shared by patients, while the white bars indicate normal allele. High hyperopia in the family is transmitted as an autosomal dominant trait, and the gene responsible for high hyperopia is mapped to chromosome 11p12–q13.3 between D11S935 and D11S4196. Asterisks indicates the samples which have been sent for WES

One hundred and twenty-two probands with high hyperopia and their available family members, including a large family with 14 affected individuals across four generations (Family ZOC710536), were recruited from our outpatient clinic at the Zhongshan Ophthalmic Center as part of our ongoing program for the collection of genomic DNA to investigate hereditary eye diseases. Written informed consent was obtained from the participating individuals or their guardians prior to the study, in compliance with the Declaration of Helsinki and the Guidance of Sample Collection of Human Genetic Diseases (863-Plan) by the Ministry of Public Health of China. This study has been approved by the Institutional Review Board of the Zhongshan Ophthalmic Center. The disease in family ZOC710536 was likely transmitted as an autosomal dominant trait (Fig. 1). The diagnosis of high hyperopia was based on the following criteria as previously described (Carricondo et al. 2018; Fuchs et al. 2005; Xu et al. 2016): (1) bilateral cycloplegic sphere refraction ≥ + 5.00 D based on the Optometric Clinical Practice Guidelines for the Care of the Patient with Hyperopia (Association 2006), or ocular axial length < 21 mm (Carricondo et al. 2018; Fuchs et al. 2005); (2) corneal diameter in the normal range (≥ 11 mm) (Carricondo et al. 2018); (3) no systemic hereditary disease or ocular disease, including retinal degeneration or developmental anomalies of the iris, lens, choroid or optic disc. Available subjects usually received routine ophthalmological examinations, including visual acuity testing, slit-lamp examination and direct ophthalmoscopic visualization. Refractive errors were measured using an autorefractometer after cycloplegia, and ocular axial length was measured using an optical biometer, as described in our previous study (Xu et al. 2016).

### Mapping and identification of the novel causative gene

For the largest family in this study with high hyperopia (ZOC710536), parallel genome-wide linkage scans, whole-exome sequencing (WES), and whole-genome sequencing of genomic DNA were carried out as described in our previous study (Xiao et al. 2016). WES alone was also performed on genomic DNA from 121 probands of additional families with high hyperopia. Sanger sequencing was used to confirm potential pathogenic mutations.

Briefly, a genome-wide linkage scan was initially conducted on genomic DNA from 16 of the 19 individuals in family ZOC710536 (excluding IV:1, IV:3, and V:3; Fig. 1) using 400 5′-fluorescently labeled microsatellite markers according to panels 1–28 of the ABI PRISM linkage Mapping Set Version 2 as previously described (Zhang et al. 2005). Two-point linkage analysis was performed using the MLINK program of the FASTLINK implementation of the LINKAGE program package (Lathrop and Lalouel 1984; Schaffer et al. 1994). The high hyperopia in the family was characterized as an autosomal dominant trait with complete penetrance and a disease allele frequency of 0.0001. Fine mapping of a candidate locus was carried out on all 19 individuals in the family using an M13-tailed primer PCR method to genotype additional markers (Barkley et al. 2007). Haplotypes were generated using the Cyrillic 2.1 program (Cyrillic Software, Wallingford, Oxfordshire OX10 8BA) and confirmed by inspection. The criteria for establishing linkage have been previously described (Lander and Kruglyak 1995).

Meanwhile, WES was performed on genomic DNA from four affected individuals (III:5, III:9, IV:2, and V:1 in Fig. 1) in family ZOC710536, as well as from 121 probands in other additional families with high hyperopia, with an Agilent SureSelect Human All Exon Enrichment Kit V4 (Santa Clara, CA, USA) (51189318 base pairs) array on an Illumina HiSeq 2000 101PE (San Diego, CA, USA) as described in our previous study (Sun et al. 2015). Variants detected by WES were initially filtered by multistep bioinformatics analysis: the stages were as follows: (1) excluding variants in noncoding regions as well as synonymous variants that did not affect splice sites according to the Berkeley Drosophila Genome Project [BDGP; http://www.fruitfly.org/ (in the public domain)]; (2) excluding variants with minor allele frequency (MAF) ≥ 1%; (3) excluding variants not shared by four affected individuals in the largest family, ZOC710536; and (4) searching for unique variants in genes with similar mutations in 121 additional families with high hyperopia. WES data from the Exome Aggregation Consortium (ExAC) database and our in-house WES data from 3280 unrelated probands with different forms of inherited eye diseases served as reference. Sanger dideoxy sequencing was used to confirm potential pathogenic variants and to evaluate their cosegregation in family members. Whole-genome sequencing on two Individuals (IV:7 and IV:9 in Fig. 1) was carried out using a commercial service to test for mutations in the linkage interval that had been missed by WES.

### Knockdown of *myrf* in zebrafish

Wild-type AB zebrafish (*Danio rerio*) embryos were obtained from Sun Yat-sen University. Zebrafish embryos were fertilized in vitro at 28.5 °C, maintained on a 14-h light:10-h dark cycle, and staged in hours post-fertilization (hpf) or days post-fertilization (dpf). Two morpholino oligonucleotides (MOs) were purchased from Gene Tools, LLC (Corvallis, OR, USA), including an antisense MO targeting translation initiation (ATG) of *myrf* (*myrf* MO, 5′-CAATCACGGTTTCCATTCTAGCGGC-3′) and a standard control MO (std MO, 5′-CCTCTTACCTCAGTTACAATTTATA-3′). Both of the MOs were dissolved in distilled water and microinjected into the egg yolks of zebrafish embryos in the one-cell stage. To test the efficiency of *myrf* MO, we amplified the complementary sequence of *myrf* MO using primers crossing the MO sequence, designed in Primer3 (S1 Table) and inserted in-frame before the green fluorescent protein (GFP) coding sequence of the pEGFPN1 vector.

For the rescue experiment, the full-length coding sequence of *myrf* in zebrafish was amplified by PCR using cDNA from wild-type zebrafish at 72 hpf as the template and cloned into the pCS2+ vector (primers are listed in S1 Table). The *myrf* mRNA was synthesized using the mMESSAGE mMACHINE™ SP6 kit (Invitrogen AM1340) and pCS2-*myrf* plasmid as a template. The mRNA was diluted to 106 pg and coinjected with *myrf* MO into the egg yolks during the one-cell stage. Zebrafish larvae were anesthetized with 0.03% tricaine at 72 hpf to observe the phenotypes. The ocular phenotypes of zebrafish larvae were analyzed at 72 hpf by light microscopy. The eye sizes of the larvae, defined as the distance from the nasal side to the temporal side of the eye, were measured using ImageJ software and were compared among different groups by a one-way ANOVA by SPSS23.0 software.

Zebrafish larvae at 5 dpf were fixed with 4% paraformaldehyde (PFA) for 16 h at 4 °C, equilibrated in 30% sucrose solution for at least 8 h, embedded in OCT (optimal cutting temperature compound) tissue freezing medium, and frozen. Frozen sections (10 μm) were produced using a Leica Cryostat Microtome (CM1950). Slides were processed for antigen retrieval at 98 °C for 30 min and incubated with the following primary antibodies for 16 h at 4 °C: anti-Hu C/D (1:100; Invitrogen; A-21271), anti-Pax6 (1:500; Covance; PRB-278P-100), anti-PKC α (1:500; Santa Cruz Biotechnology; sc-208), anti-glutamine synthetase (GS; 1:1000; BD Biosciences; 610518), anti-recoverin (1:500; Millipore; AB5585), and anti-Rho 1D4 (1:500; Abcam; ab5417). Sections were incubated in secondary antibodies, including Alexa Fluor 488-conjugated goat anti-mouse IgG antibody (1:500; Cell Signaling Technology 4408) and Alexa Fluor 555-conjugated goat anti-rabbit IgG antibody (1:500; Cell Signaling Technology 4413), for 2 h at room temperature. Images were collected with a Nikon C2 confocal microscope and a Zeiss 788 confocal microscope.

## Results

### Identification of the genetic locus in family ZOC710536

**Fig. 2:**
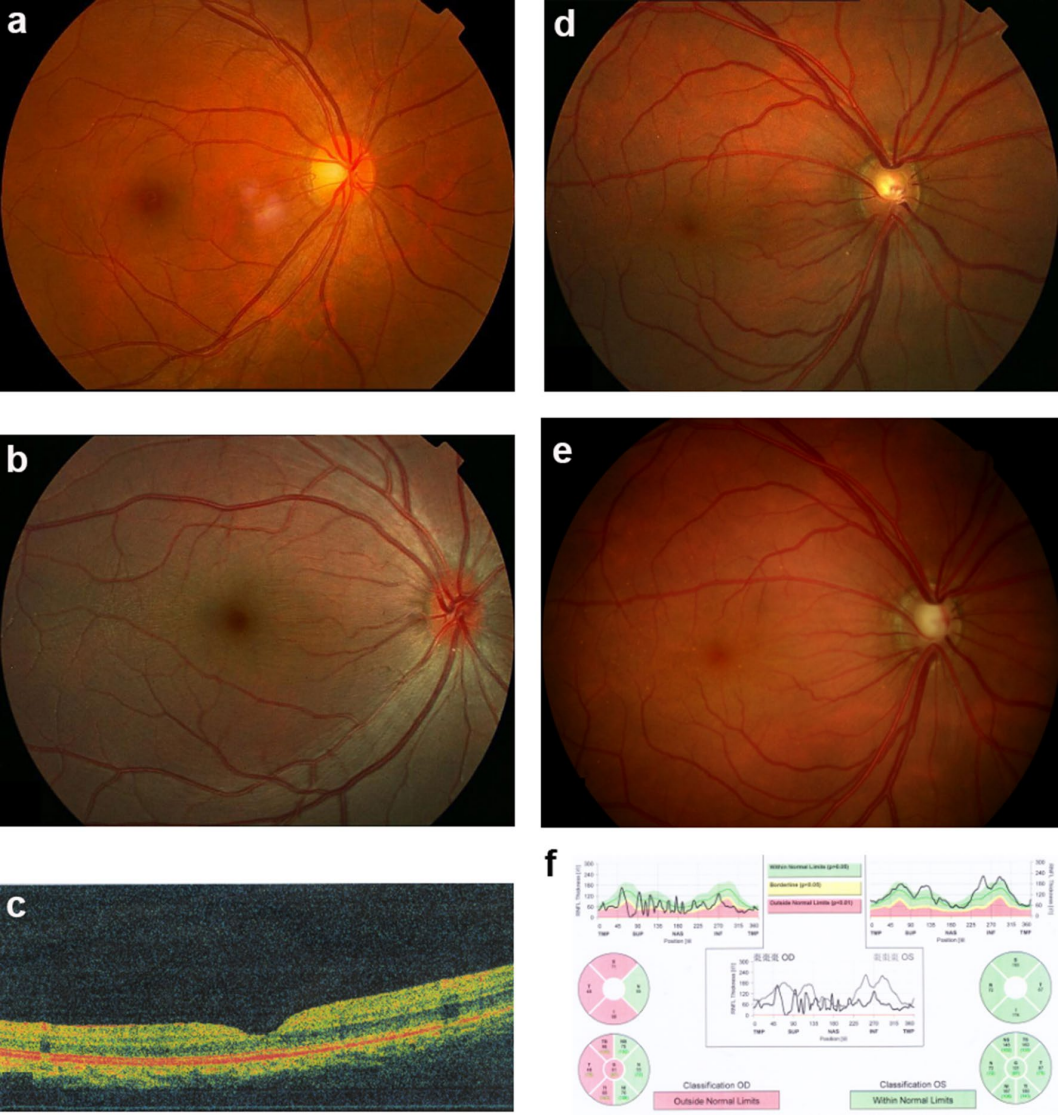
Fundus changes associated with high hyperopia in family ZOC710536. **a** Normal fundus of an unrelated normal control. **b** Fundus photograph of V:1. The right eye for the affected individual V:1 in Fig. 1 at 6 years old with refraction of + 12D/OS and + 12D/OS, and an axial length of 17.49 mm/OD and 17.53 mm/OS. Fundus change is typical for high hyperopia with a relatively normal fovea. **c** OCT scan of V:1. The right eye for the affected individual V:1 in Fig. 1 at 6 years old with refraction of + 12D/OS and + 12D/OS and an axial length of 17.49 mm/OD and 17.53 mm/OS. **d** Fundus photos of IV: 7 at 29 years old. The right eye for the affected individual IV:7 in Fig. 1 at 29 years old, with refraction of + 10.00 DS/OD and + 10.00 DS/OS and an axial length of 17.6 mm/OD and 17.47 mm/OS. **e** Fundus photos of IV: 7 at 38 years old. The right eye for the individual IV:7 in Fig. 1 at 38 years old, 2 weeks after the onset of angle-closure glaucoma in the right eye (with IOP 43 mmHg before treatment). An enlarged optic disc was observed compared with the fundus photograph in D. **f** Heidelberg retina tomograph (HRT) results for the affected individual IV:7 from Fig. 1. The HRT result in F demonstrated partial loss of the retinal ganglion cell layer in the temporal half of the retina in the right eye. *OD* right eye, *OS* left eye

In family ZOC710536, high hyperopia is transmitted as an autosomal dominant trait (Fig. 1), with male-to-male transmission across at least four generations. Clinical data and genomic DNA were obtained from 19 individuals, including 14 affected and 5 unaffected members, comprising 11 males and 8 females. Refraction in the 14 affected individuals ranged from + 5.00 to + 12.00 diopters, while refraction in 5 unaffected members ranged from 0 to − 3.00 diopters. Ocular axial length was available in three affected members: 17.60 mm/OD and 17.47 mm/OS for IV:7, 17.49 mm/OD and 17.53 mm/OS for V:1, and 18.62 mm/OD and 18.76 mm/OS for V:2. All 19 family members had clear corneas with corneal diameters between 11 and 12 mm. Affected individuals had fundus changes typical of high hyperopia (Fig. 2). Strabismus was not observed in any of the 14 affected members. Angle-closure glaucoma in the right eye, with maximum intraocular pressure of 43 mmHg, had recently developed in one individual (IV:7) at the age of 38 years, 9 years after collection of the family data.

An initial genome-wide linkage scan was performed on 16 of the 19 family members (excluding IV:1, IV:3, and V:3; Fig. 1), to save funds and time (two rows of 16 samples fit well in our ABI3130 sequencer). Two-point linkage analysis revealed only one candidate locus on chromosome 11, with maximum LOD scores of 3.46, 3.17, and 2.72 for markers D11S4191, D11S987, and D11S905, respectively. Fine mapping with all 19 individuals (including IV:1, IV:3, and V:3 in Fig. 1) and additional markers confirmed the locus on chromosome 11, where ten closely spaced microsatellite markers in the region generated positive LOD scores, with D11S987, D11S1889, and D11S4191 yielding LOD scores of 4.68, 4.21, and 4.07, respectively, at theta = 0 (Table 1). Haplotype construction demonstrated that all patients in the family shared a haplotype block of D11S4102-D11S1360-D11S905-D11S1313-D11S4191-D11S4076-D11S4205-D11S1889-D11S987-D11S4113. The proximal and telomeric boundaries were indicated by recombination between D11S935 and D11S4102 in individuals III:7, III:9, IV:5, IV:6, IV:7, V:1, V:2, and V:3 as well as recombination between D11S4113 and D11S4196 in individual IV:5. The data strongly support that the disease gene is situated in a 27.1 cM region between D11S935 and D11S4196 on chromosome 11p12–q13.3 (Fig. 1).

**Table 1:** Two-point Lod scores between high hyperopia in the Chinese family and markers around 11p12–11q13.3

Markers	Position		Lod score at theta =						
	cM^a^	Mb^b^	0.00	0.01	0.05	0.10	0.20	0.30	0.40
D11S935	49.6	36.02	− 1.52	0.75	1.29	1.37	1.13	0.72	0.28
D11S4102	51.5	36.78	1.29	1.25	1.11	0.92	0.55	0.22	0.03
D11S1360	54.2	40.23	1.65	1.64	1.57	1.41	0.99	0.54	0.18
D11S905	55.7	40.97	2.72	2.68	2.51	2.27	1.73	1.13	0.51
D11S1313	63.2	56.23	2.64	2.60	2.43	2.19	1.65	1.05	0.44
D11S4191	63.4	60	4.07	3.99	3.70	3.31	2.49	1.61	0.71
D11S4076	64.9	61.36	2.40	2.38	2.25	2.04	1.50	0.87	0.26
D11S4205	67.4	63.18	1.20	1.17	1.05	0.90	0.60	0.31	0.09
D11S1889	70.8	67.31	4.21	4.13	3.80	3.38	2.49	1.55	0.63
D11S987	NA	67.89	4.68	4.60	4.27	3.83	2.91	1.90	0.84
D11S4113	71.6	68.77	3.71	3.65	3.43	3.12	2.42	1.64	0.81
D11S4196	76.7	NA	− inf	2.69	3.07	2.96	2.39	1.61	0.74
D11S1314	77.5	72.32	− inf	2.14	2.55	2.46	1.94	1.24	0.49

^a^Genethon

^b^The position refers to human genome (Build 37.2 version) Chr11 Primary_Assembly

### Identification of a novel gene for high hyperopia in family ZOC710536

**Fig. 3:**
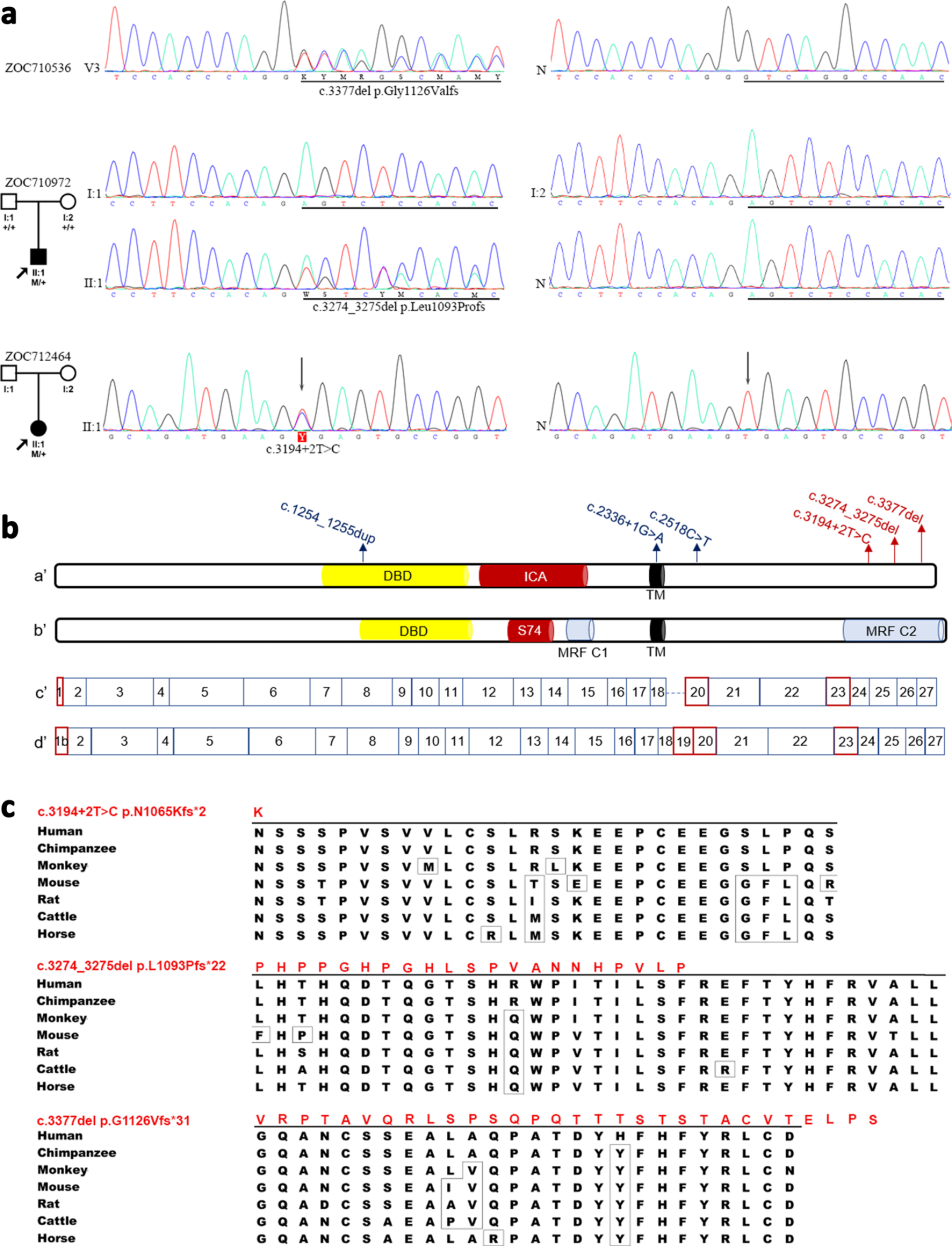
Sequence chromatography, distribution, and conservation of the three variants in MYRF. **a** Sequence chromatography from Sanger sequencing. Variants in MYRF identified by exome sequencing were further confirmed with Sanger sequencing and then validated in available family members. Pedigrees of ZOC710972 and ZOC712464 are shown in the left column. Under each individual, + indicates a wild-type allele, and M indicates a mutant allele. The sequences with or without variants detected in patients with high hyperopia, available parents, as well as normal controls are shown in the middle and right columns. **b** Distribution of variants in *MYRF* and exon coverage corresponding to the predicted protein schematic of MYRF. Mutations associated with high hyperopia are indicated in red, while mutations associated with cardiac and urogenital anomalies are indicated in blue. **a**′, **b**′ Predicted schematic of MYRF referring to the NM_013279.3 and NM_001127392.2 isoforms, respectively (Pinz et al. 2018). *DBD* DNA-binding domain, *ICA* intramolecular chaperone autoprocessing domain, *TM* transmembrane domain, *S74* peptidase S74, chaperone of endosialidase domain, *MRF C* myelin gene regulatory factor C-terminal domain. **c**′, **d**′ Exon coverage of MYRF, referring to the NM_013279.3 and NM_001127392.2 isoforms, respectively. Exons differing between the two isoforms are indicated in red. **c** Protein sequence alignment of seven MYRF orthologs. The residues differing from the human sequence are indicated with boxes. The three variants and the associated new residue sequences resulted from them are shown above and indicated with red

For parallel WES of genomic DNA from four affected individuals (III:5, III:9, IV:2, and V:1) in family ZOC710536, the average throughput depth, mean read depth, and coverage of the target regions were 195.5X, 49.9X, and 89.1% for III:5; 228.8X, 63.6X, and 90.2% for III:9; 191.7X, 57.3X, and 90.0% for IV:2; and 203.3X, 66.7X, and 90.1% for V:1. Comparison of sequencing data among the four individuals revealed that 14 rare variants affecting coding residues were shared by all four patients, including 12 missense mutations, one non-frameshift insertion, and one frameshift deletion. Of the 14, five were located inside the linkage interval on chromosome 11p12–q13.3. All but one of the 14 were excluded as candidate mutations for high hyperopia in the family after comparison of the 14 variants with data on the corresponding genes from ExAC and our in-house data from 3280 unrelated individuals. The remaining one is a novel variant located at chr11:61553536 in exon 27 (the last exon) of the myelin regulatory factor (*MYRF*) gene (HGNC ID: 1181; Entrez Gene: 745; Ensembl: ENSG00000124920; OMIM: 608329), also named C11ORF9 or KIAA0954, located at 11q12.2 [genomic location: chr11: 61,752,617–61,788,518 (GRCh38/hg38), or chr11: 61,520,121–61,555,990 (GRCh37/hg19)] that is situated inside the linkage interval. This potential pathogenic variant, a c.3377delG (p.Gly1126Valfs*31) based on NM_001127392, is predicted to result in the loss of the last 26 of the 1151 residues of MYRF and the addition of 30 new residues. Such variants are classified as loss-of-function (LoF) mutations, a category that includes nonsense, frameshift, splicing acceptor, and splicing donor variants; these mutations are extremely rare in *MYRF*, and the gene is, therefore, considered extremely LoF intolerant (pLI = 1.0) based on the ExAC database (http://exac.broadinstitute.org/gene/ENSG00000124920, Sep 25th 2018). This variant is not present in ExAC or in any additional families among the 3280 with WES data. The c.3377delG mutation was further confirmed by Sanger sequencing (Fig. 3) and was completely cosegregated with high hyperopia in family ZOC710536.

### Detection of *MYRF* mutations in two additional families with high hyperopia

Apart from the novel mutation identified in the large family mentioned above, two additional novel truncation variants in *MYRF* were detected in 2 of the other 121 probands with high hyperopia based on WES. Of the two, a c.3274_3275del (p. Leu1093Profs*22) mutation in exon 25 was detected in a 6-year-old boy with high hyperopia and axial lengths of 20.90 mm and 20.68 mm for the right and left eyes, respectively. The c.3274_3275del mutation was a de novo mutation that was not present in the boy’s parents (Fig. 3), who had normal visual acuity and normal axial length. The remaining one, a c.3194+2T>C predicted to result in loss of the splicing donor in intron 24, was detected in a 40-year-old singleton female with high hyperopia and axial lengths of 19.73 mm and 19.83 mm. This individual had recently developed angle-closure glaucoma of both eyes, with a highest intraocular pressure of 42 mmHg.

Similar to the c.3377delG mutation, the c.3274_3275del and c.3194+2T>C mutations were confirmed by Sanger sequencing (Fig. 3) and were present in neither the ExAC database nor the rest of the 3280 probands from our in-house WES data. Except for the three novel truncation mutations described in the above three unrelated families, no additional truncation mutations were found in WES data from the 3280 probands with other forms of genetic eye diseases. A number of missense variants were also detected in *MYRF* by WES, but they were distributed in patients with different genetic eye diseases, as well as controls, without enrichment in any special subgroup.

### Knockdown of *myrf* resulted in small eye size

**Fig. 4:**
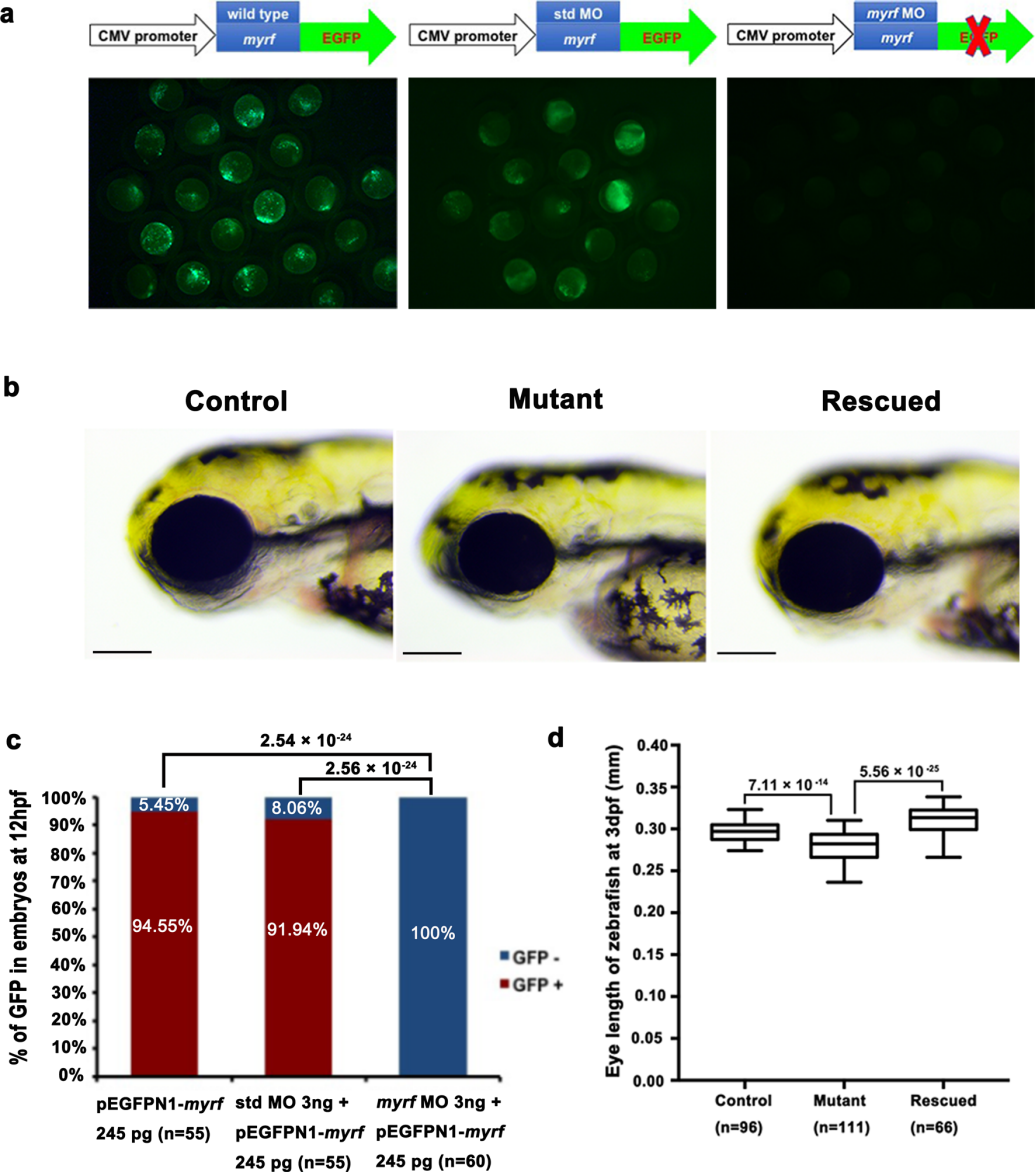
Phenotype of *myrf* knockdown zebrafish. **a** The efficiency of *myrf* MO knockdown of the *myrf* gene. Strong GFP signals were present in the left and middle images, which represent injection with *myrf*-pEGFPN1 plasmid alone and coinjected of *myrf*-pEGFPN1 plasmid with std MO, respectively. No GFP signal was detected in the embryos coinjected with *myrf*-pEGFPN1 plasmid and *myrf* MO, as shown in the right image. **b** Embryos with *myrf* knockdown showed a phenotype of small eye size (middle image) compared to the std MO-injected embryos (left image). This phenotype could be rescued by coinjection of *myrf* mRNA (right image). Control is larvae injected with 3 ng of std MO at 72 hpf. Mutant is larvae injected 3 ng of myrf MO at 72 hpf. Rescue is larvae coinjected with 3 ng of myrf mRNA and 106 pg of myrf MO at 72 hpf. **c** Proportions of embryos with GFP^+^ or GFP^−^ treated with 245 pg pEGFPN1-*myrf*, co-injection of 3 ng std MO with 245 pg of pEGFPN1-*myrf* and coinjection of 3 ng *myrf* MO with 245 pg of pEGFPN1-myrf. The p values by the Chi-squared test are shown above. **d** Box plot of the median and deviations of eye size in three groups. The *p* values by one-way ANOVA are shown above

To further analyze the function of *myrf* in eye development, we developed a *myrf* knockdown zebrafish model by injecting of *myrf* MO into zebrafish eggs to block translation of the gene. The *myrf* MO showed high efficiency in knocking down expression of the *myrf* gene in zebrafish, as no GFP signal was detected in embryos at 12 hpf after coinjection of 254 pg of *myrf*-pEGFPN1 plasmid and 3 ng of *myrf* MO coinjected embryos, while a strong GFP signal was detected in embryos injected with 254 pg *myrf*-pEGFPN1 alone or in conjunction with 3 ng of std MO (Fig. 4a, c). The mean (± standard error, SE) was 296.10 (± 11.68) μm of 96 larvae injected with 3 ng of std MO at 72 hpf and 279.07 (± 18.03) μm of 111 larvae injected with 3 ng of *myrf* MO at 72 hpf, respectively. A significant reduction in eye size was detected in larvae injected with 3 ng of *myrf* MO at 72 hpf than larvae injected with 3 ng of std MO (*p* = 7.11 × 10^−14^) (Fig. 4b, d). No other obvious bodily abnormalities were found in the zebrafish larvae. To further confirm the specificity of *myrf* MO against *myrf*, we synthesized *myrf* mRNA with a 3-nt silent mutation in the *myrf* MO targeting sequence and coinjected it with *myrf* MO for the rescue experiments. Indeed, the mean (± SE) was 310.25 (± 14.81) μm of 66 larvae coinjected with *myrf* mRNA and *myrf* MO at 72 hpf, which significantly larger than that of larvae injected with *myrf* MO alone (*p* = 5.56 × 10^−25^) (Fig. 4b, d). These results suggested that the phenotype of small eye size in *myrf* morphants was specifically caused by *myrf* gene knockdown in zebrafish. Taken together, *myrf* MO knockdown and the rescue experiments illustrated that *myrf* played an important role in zebrafish eye development.

**Fig. 5:**
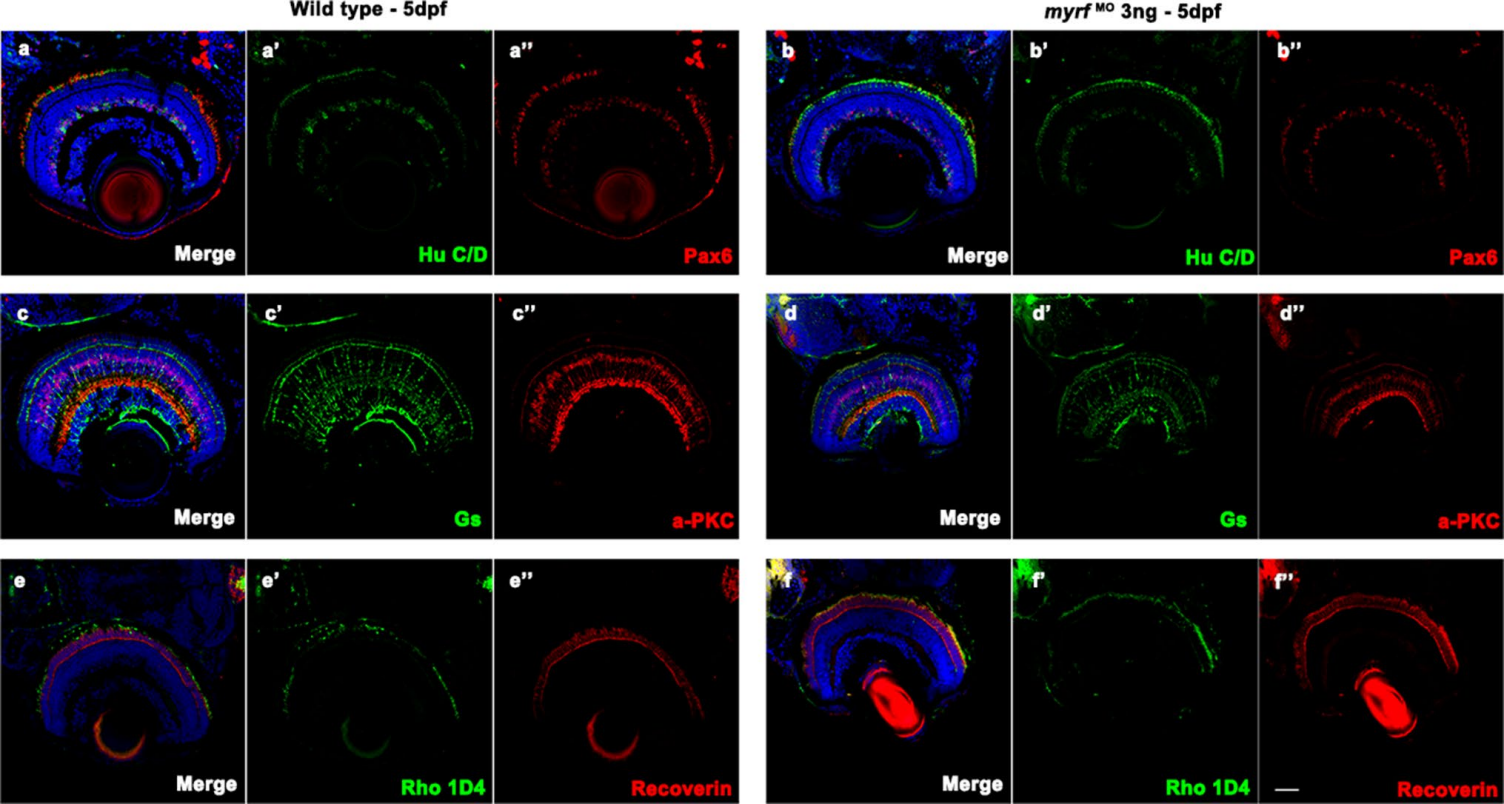
Immunostaining images of labels for different retinal cells in zebrafish at 5 dpf. Frozen sections from wild-type larvae (**a**–**a**″, **c**–**c**″, and **e**–**e**″) and *myrf* MO-injected larvae (**b**–**b**″, **d**–**d**″, and **f**–**f**″). Zebrafish larvae were labeled with Hu C/D (ganglion cell and amacrine cell marker, green, **a**′ and **b**′), Pax 6 (ganglion and amacrine precursor cell marker, red, **a**′′ and **b**′′), Gs (Müller cell marker, green, **c**′ and **d**′), PKCα (bipolar cell marker, red, **c**′′ and **d**′′), Rho 1D4 (long double cone outer segment marker, green, **e**′ and **f**′), recoverin (cone bipolar cell marker, red, **e**″ and **f**″), and DAPI (nuclei, blue). No differences were detected in retinal development between wild-type larvae and *myrf* morphants. Scale bars represent 100 μm

Since a phenotype of small eye size was detected in *myrf* morphants, we wanted to detect whether *myrf* affected retinal cell development. Immunofluorescent staining for different retinal cell labels showed that all of the ganglion cells, amacrine cells, bipolar cells, Müller cells, cone bipolar cells, and long double cone outer segments in the retina developed normally in *myrf* morphants compared with wild-type larvae at 5 pdf (Fig. 5).

## Discussion

In this study, autosomal dominant high hyperopia in a large Chinese family was mapped to chromosome 11p12–q13.3 between D11S935 and D11S4196. The exclusion of other regions in the genome, the maximum positive LOD score of 4.68 together with haplotype support, the detection of a novel truncation mutation in *MYRF* that completely segregated with high hyperopia in the family, the identification of two other novel truncation mutations in the same gene in two additional families, the extreme rarity of truncation mutations in this gene, and the exclusion of potential pathogenic mutations in other genes, and the results from *myrf* knockdown zebrafish all support the case that truncation mutations in the C-terminal portion in *MYRF* are responsible for autosomal dominant high hyperopia in these families.

Previously, an autosomal dominant form of nanophthalmos (NNO1) with high hyperopia and angle-closure glaucoma was mapped to chromosome 11p between D11S905 and D11S987, with a maximum LOD score of 5.92 (Othman et al. 1998). The linkage interval of NNO1 overlaps with the linkage interval identified in our current study. However, the *MYRF* gene is located outside the linkage interval of NNO1; thus, the gene responsible for NNO1 has yet to be identified. Therefore, *MYRF*-associated high hyperopia should be considered to have a separate locus. Nevertheless, *MYRF*-associated high hyperopia and NNO1 share common clinical features, such as high hyperopia, short axial length, and angle-closure glaucoma. It would be interesting to know the sequence variations of *MYRF* in the affected individuals of the family with NNO1. Meanwhile, the association of angle-closure glaucoma with *MYRF* mutations is of special interest for future studies because the molecular basis of angle-closure glaucoma is largely unknown, although this condition is a common cause of blindness, especially in Asian populations (Sun et al. 2017).

The initial *MYRF* full-length cDNA was cloned from an eye-tissue-specific cDNA library and was expressed in the retina and several other tissues (Stohr et al. 2000). Updated annotation of the *MYRF* gene shows two variant transcripts, of which transcript variant 2 is longer, with 27 exons coding for 1151 residues, while transcript variant 1 has 26 exons coding for 1111 residues (https://www.ncbi.nlm.nih.gov/nuccore/NC_000011.10?from=61752617&to=61788518&report=genbank). Transcript variant 1 has a different 5′ terminal exon, lacks an exon corresponding to exon 19 in transcript variant 2, and has different splicing acceptors for exons 19 and 22 (corresponding to exons 20 and 23 of transcript variant 2). All three novel truncation mutations identified in the current study are located in exons shared by the two variant transcripts (splicing donor of intron 24, exon 25, and exon 27). *MYRF* is widely expressed in mouse tissue (Nakayama et al. 2018) and is enriched in the central nervous system (Cahoy et al. 2008; Emery et al. 2009). This gene encodes an endoplasmic reticulum membrane-anchored transcription factor that is specifically expressed in oligodendrocytes and is critical for oligodendrocyte differentiation, as well as for the generation and maintenance of myelination in the central nervous system (Bujalka et al. 2013; Duncan et al. 2017; Emery et al. 2009; Kim et al. 2017; Koenning et al. 2012; Li and Richardson 2016). Through autocleavage, the nuclear-targeted N-terminal portion, which contains a DNA-binding domain, is separated from the C-terminal portion and then translocated from the endoplasmic reticulum to the nucleus, where it directly binds the enhancer regions of genes underlying myelination in oligodendrocytes (Bujalka et al. 2013; Li et al. 2013). *MYRF* is also indispensable for synaptic plasticity in *C. elegans* (Meng et al. 2017). The role of the C-terminal region is still unknown and may potentially involve MYRF folding, localization, cleavage, or regulation (Bujalka et al. 2013). Interestingly, all three novel truncation mutations identified in the current study are located in the C-terminal region of MYRF (Fig. 3).

Thus far, mutations in *MYRF* have been reported to have possible associations with several diseases, although evidence is not confirmatory from a genetic point of view. Missense variants in *MYRF* have been reported to have a significant association (*p* < 0.05) with late-onset Alzheimer’s disease (Vardarajan et al. 2018). Two SNPs near *MYRF* have been reported to associate with blood systolic pressure based on genotyping of approximately 200,000 SNPs in 1947 black South African individuals (rs11230796, *p* = 2.16 × 10^−7^; rs400075, *p* = 2.88 × 10^−7^) (Hendry et al. 2018). In addition, three heterozygous de novo truncation variants in *MYRF* have been detected in three singleton males with cardiac and urogenital anomalies (Chitayat et al. 2018; Pinz et al. 2018). Furthermore, a heterozygous c.1208A>G (p.Gln403Arg) mutation has been detected in two families with encephalopathy with reversible myelin vacuolization, with incomplete penetrance (Kurahashi et al. 2018). Mice with homozygous conditional knockout of *MYRF* in oligodendrocytes demonstrate severe tremors and ataxia from postnatal day 11, subsequently develop seizures, and die during the third postnatal week (Emery et al. 2009). However, the phenotype of mice with heterozygous conditional knockout has not been described (Emery et al. 2009). The nature of the variants described in our current study is different from most of what is described in the above-mentioned reports, except for the three males with cardiac and urogenital anomalies, and the location of variants is also different. Our patients with high hyperopia do not present systemic signs or symptoms suggesting brain, cardiac or urogenital defects. All the three truncation variants identified in families with high hyperopia in the current study are located at the C-terminal of MYRF and absent in gnomAD database. In contrast, the other truncation variants in MYRF are all located upstream of the three variants, including three variants identified in patients with cardiac and urogenital anomalies (Table S2, Fig. 3b) and additional 19 LoF variants in gnomAD database. Therefore, it might be a gain-of-function model by the three variants in patients with high hyperopia in the current study, especially one (c.3377delG) is located in the last exon which could escape the nonsense-mediated decay. The phenotypes associated with different *MYRF* variants may need further clarification, although our current study provides the strongest genetic evidence to date in support of the association between *MYRF* truncation mutation and high hyperopia.

A disease called myelinated retinal nerve fibers (MRNF), consisting of abnormal myelination with ectopic oligodendrocyte-like cells in the retina, is a common congenital anomaly that is observed in approximately 1% of all eyes (Nangia et al. 2014; Tarabishy et al. 2007). This condition is frequently associated with ipsilateral myopia or hyperopia (Lee and Salchow 2008; Nangia et al. 2014; Tarabishy et al. 2007). A recent study demonstrated a significant association between MRNF and hyperopia based on an analysis of 52 adult eyes with MRNF in rural Central India (Nangia et al. 2014). It is still unknown whether variants in *MYRF* contribute to MRNF, although *MYRF* plays a critical role in myelination. *SOX10* regulates and cooperates with *MYRF* (Hornig et al. 2013; Lopez-Anido et al. 2015). Mutations in *SOX10* cause Waardenburg syndrome, which is characterized by developmental anomalies of the eye (Pingault et al. 1998, 2010). These data also imply that mutations in *MYRF* may contribute to developmental anomalies of the eye, including high hyperopia with short axial length.

The molecular mechanism underlying high hyperopia development with *MYRF* truncation mutations is not yet known. Retinal progenitor neuron cells and specifically expressed functional genes in these cells may not only play roles in defining specific ocular structure or biological functions but also participate in the developmental process of eye development. Further study on these truncation mutations in the C-terminal portion of *MYRF* may not only reveal the molecular pathway contributing to high hyperopia but also provide a valuable avenue for investigating the role of MYRF C-terminal portion.

In summary, high hyperopia in a large family is mapped to chromosome 11p12–q13.3, a locus overlapping with NNO1, by genome-wide linkage analysis. WES and whole-genome sequencing identified novel truncation mutations in the C-terminal portion of MYRF, located inside the linkage interval of high hyperopia but outside the linkage interval of NNO1, in three unrelated families including the family whose mutation is mapped to this region. Informatic approaches using mutation prediction and a mutation database, as well as an experimental approach using knockdown of *myrf* in zebrafish, further support linkage mapping and mutation identification. Our findings may provide useful clues to further elucidate the function of the critical myelin regulatory factor MYRF as well as the molecular pathogenesis of high hyperopia and its associated angle-closure glaucoma.

## Electronic supplementary material

Below is the link to the electronic supplementary material.

Supplementary material 1 (DOCX 19 kb)

## References

[CR1] Abouzeid H, Li Y, Maumenee IH, Dharmaraj S, Sundin O (2006). A G1103R mutation in CRB1 is co-inherited with high hyperopia and Leber congenital amaurosis. Ophthalmic Genet.

[CR2] Aldahmesh MA, Nowilaty SR, Alzahrani F, Al-Ebdi L, Mohamed JY, Rajab M, Khan AO, Alkuraya FS (2011). Posterior microphthalmos as a genetically heterogeneous condition that can be allelic to nanophthalmos. Arch Ophthalmol.

[CR3] Altintas AK, Acar MA, Yalvac IS, Kocak I, Nurozler A, Duman S (1997). Autosomal recessive nanophthalmos. Acta Ophthalmol Scand.

[CR4] Association AO (2006) Optometric Clinical Practice Guideline: Care of the patient with hyperopia. http://www.aoa.org/documents/CPG-16.pdf

[CR5] Awadalla MS, Burdon KP, Souzeau E, Landers J, Hewitt AW, Sharma S, Craig JE (2014). Mutation in TMEM98 in a large white kindred with autosomal dominant nanophthalmos linked to 17p12–q12. JAMA Ophthalmol.

[CR6] Barkley NA, Dean RE, Pittman RN, Wang ML, Holbrook CC, Pederson GA (2007). Genetic diversity of cultivated and wild-type peanuts evaluated with M13-tailed SSR markers and sequencing. Genet Res.

[CR7] Bifari IN, Elkhamary SM, Bolz HJ, Khan AO (2016). The ophthalmic phenotype of IFT140-related ciliopathy ranges from isolated to syndromic congenital retinal dystrophy. Br J Ophthalmol.

[CR8] Bujalka H, Koenning M, Jackson S (2013). MYRF is a membrane-associated transcription factor that autoproteolytically cleaves to directly activate myelin genes. PLoS Biol.

[CR9] Cahoy JD, Emery B, Kaushal A (2008). A transcriptome database for astrocytes, neurons, and oligodendrocytes: a new resource for understanding brain development and function. J Neurosci.

[CR10] Carricondo PC, Andrade T, Prasov L, Ayres BM, Moroi SE (2018). Nanophthalmos: a review of the clinical spectrum and genetics. J Ophthalmol.

[CR11] Chitayat D, Shannon P, Uster T, Nezarati MM, Schnur RE, Bhoj EJ (2018). An additional individual with a de novo variant in myelin regulatory factor (MYRF) with cardiac and urogenital anomalies: further proof of causality: comments on the article by Pinz et al.. Am J Med Genet A.

[CR12] den Hollander AI, Koenekoop RK, Mohamed MD (2007). Mutations in LCA5, encoding the ciliary protein lebercilin, cause Leber congenital amaurosis. Nat Genet.

[CR13] Dirani M, Chamberlain M, Shekar SN, Islam AF, Garoufalis P, Chen CY, Guymer RH, Baird PN (2006). Heritability of refractive error and ocular biometrics: the Genes in Myopia (GEM) twin study. Invest Ophthalmol Vis Sci.

[CR14] Duncan GJ, Plemel JR, Assinck P (2017). Myelin regulatory factor drives remyelination in multiple sclerosis. Acta Neuropathol.

[CR15] Emery B, Agalliu D, Cahoy JD, Watkins TA, Dugas JC, Mulinyawe SB, Ibrahim A, Ligon KL, Rowitch DH, Barres BA (2009). Myelin gene regulatory factor is a critical transcriptional regulator required for CNS myelination. Cell.

[CR16] Erdol H, Kola M, Turk A, Akyol N (2008). Ultrasound biomicroscopy and OCT findings in posterior microphthalmos. Eur J Ophthalmol.

[CR17] Fledelius HC, Fuchs HJ, Rosenberg T (2004). Oculometric characteristics of extreme hypermetropia in two faroese families. Optom Vis Sci.

[CR18] Fuchs J, Holm K, Vilhelmsen K, Rosenberg T, Scherfig E, Fledelius HC (2005). Hereditary high hypermetropia in the Faroe Islands. Ophthalmic Genet.

[CR19] Gal A, Rau I, El Matri L (2011). Autosomal-recessive posterior microphthalmos is caused by mutations in PRSS56, a gene encoding a trypsin-like serine protease. Am J Hum Genet.

[CR20] Giordano L, Friedman DS, Repka MX, Katz J, Ibironke J, Hawes P, Tielsch JM (2009). Prevalence of refractive error among preschool children in an urban population: the Baltimore Pediatric Eye Disease Study. Ophthalmology.

[CR21] Goldblum D, Mojon DS (1999). Posterior microphthalmos associated with papillomacular fold and high hyperopia. J Pediatr Ophthalmol Strabismus.

[CR22] Hammond CJ, Snieder H, Gilbert CE, Spector TD (2001). Genes and environment in refractive error: the twin eye study. Invest Ophthalmol Vis Sci.

[CR23] Helvacioglu F, Kapran Z, Sencan S, Uyar M, Cam O (2014). Optical coherence tomography of bilateral nanophthalmos with macular folds and high hyperopia. Case Rep Ophthalmol Med.

[CR24] Hendry LM, Sahibdeen V, Choudhury A, Norris SA, Ramsay M, Lombard Z, Of the A.W.I.G.s. and as members of the H.A.C (2018). Insights into the genetics of blood pressure in black South African individuals: the Birth to Twenty cohort. BMC Med Genom.

[CR25] Hornig J, Frob F, Vogl MR, Hermans-Borgmeyer I, Tamm ER, Wegner M (2013). The transcription factors Sox10 and Myrf define an essential regulatory network module in differentiating oligodendrocytes. PLoS Genet.

[CR26] Jiang D, Yang Z, Li S, Xiao X, Jia X, Wang P, Guo X, Liu X, Zhang Q (2013). Evaluation of PRSS56 in Chinese subjects with high hyperopia or primary angle-closure glaucoma. Mol Vis.

[CR27] Jonas JB, Aung T, Bourne RR, Bron AM, Ritch R, Panda-Jonas S (2017). Glaucoma. Lancet.

[CR28] Khairallah M, Messaoud R, Zaouali S, Ben Yahia S, Ladjimi A, Jenzri S (2002). Posterior segment changes associated with posterior microphthalmos. Ophthalmology.

[CR29] Khan A, Al-Saif A, Kambouris M (2004). A novel KERA mutation associated with autosomal recessive cornea plana. Ophthalmic Genet.

[CR30] Khan AO, Bifari IN, Bolz HJ (2015). Ophthalmic features of children not yet diagnosed with alstrom syndrome. Ophthalmology.

[CR31] Kim D, Choi JO, Fan C, Shearer RS, Sharif M, Busch P, Park Y (2017). Homo-trimerization is essential for the transcription factor function of Myrf for oligodendrocyte differentiation. Nucleic Acids Res.

[CR32] Klimek DL, Cruz OA, Scott WE, Davitt BV (2004). Isoametropic amblyopia due to high hyperopia in children. J AAPOS.

[CR33] Koenning M, Jackson S, Hay CM, Faux C, Kilpatrick TJ, Willingham M, Emery B (2012). Myelin gene regulatory factor is required for maintenance of myelin and mature oligodendrocyte identity in the adult CNS. J Neurosci.

[CR34] Kurahashi H, Azuma Y, Masuda A (2018). MYRF is associated with encephalopathy with reversible myelin vacuolization. Ann Neurol.

[CR35] Lander E, Kruglyak L (1995). Genetic dissection of complex traits: guidelines for interpreting and reporting linkage results. Nat Genet.

[CR36] Lathrop GM, Lalouel JM (1984). Easy calculations of lod scores and genetic risks on small computers. Am J Hum Genet.

[CR37] Lee JC, Salchow DJ (2008). Myelinated retinal nerve fibers associated with hyperopia and amblyopia. J AAPOS.

[CR38] Lee KE, Klein BE, Klein R, Fine JP (2001). Aggregation of refractive error and 5-year changes in refractive error among families in the Beaver Dam Eye Study. Arch Ophthalmol.

[CR39] Li H, Richardson WD (2016). Evolution of the CNS myelin gene regulatory program. Brain Res.

[CR40] Li H, Wang JX, Wang CY, Yu P, Zhou Q, Chen YG, Zhao LH, Zhang YP (2008). Localization of a novel gene for congenital nonsyndromic simple microphthalmia to chromosome 2q11-14. Hum Genet.

[CR41] Li Z, Park Y, Marcotte EM (2013). A Bacteriophage tailspike domain promotes self-cleavage of a human membrane-bound transcription factor, the myelin regulatory factor MYRF. PLoS Biol.

[CR42] Lopez-Anido C, Sun G, Koenning M, Srinivasan R, Hung HA, Emery B, Keles S, Svaren J (2015). Differential Sox10 genomic occupancy in myelinating glia. Glia.

[CR43] Matsushita I, Kondo H, Tawara A (2012). Novel compound heterozygous mutations in the MFRP gene in a Japanese patient with posterior microphthalmos. Jpn J Ophthalmol.

[CR44] Meng J, Ma X, Tao H (2017). Myrf ER-bound transcription factors drive *C. elegans* synaptic plasticity via cleavage-dependent nuclear translocation. Dev Cell.

[CR45] Multi-Ethnic Pediatric Eye Disease Study, G (2010). Prevalence of myopia and hyperopia in 6- to 72-month-old African American and Hispanic children: the multi-ethnic pediatric eye disease study. Ophthalmology.

[CR46] Nair KS, Hmani-Aifa M, Ali Z (2011). Alteration of the serine protease PRSS56 causes angle-closure glaucoma in mice and posterior microphthalmia in humans and mice. Nat Genet.

[CR47] Nakayama S, Yumimoto K, Kawamura A, Nakayama KI (2018). Degradation of the endoplasmic reticulum-anchored transcription factor MyRF by the ubiquitin ligase SCF(Fbxw7) in a manner dependent on the kinase GSK-3. J Biol Chem.

[CR48] Nangia V, Jonas JB, Khare A, Bhate K, Agarwal S, Panda-Jonas S (2014). Prevalence of myelinated retinal nerve fibres in adult Indians: the Central India Eye and Medical Study. Acta Ophthalmol.

[CR49] Nowilaty SR, Khan AO, Aldahmesh MA, Tabbara KF, Al-Amri A, Alkuraya FS (2013). Biometric and molecular characterization of clinically diagnosed posterior microphthalmos. Am J Ophthalmol.

[CR50] Nowilaty SR, Mousa A, Ghazi NG (2013). The posterior pole and papillomacular fold in posterior microphthalmos: novel spectral-domain optical coherence tomography findings. Ophthalmology.

[CR51] Orr A, Dube MP, Zenteno JC (2011). Mutations in a novel serine protease PRSS56 in families with nanophthalmos. Mol Vis.

[CR52] Othman MI, Sullivan SA, Skuta GL (1998). Autosomal dominant nanophthalmos (NNO1) with high hyperopia and angle-closure glaucoma maps to chromosome 11. Am J Hum Genet.

[CR53] Park SH, Ahn YJ, Shin SY, Lee YC (2016). Clinical features of posterior microphthalmos associated with papillomacular fold and high hyperopia. Clin Exp Optom.

[CR54] Pingault V, Bondurand N, Kuhlbrodt K (1998). SOX10 mutations in patients with Waardenburg–Hirschsprung disease. Nat Genet.

[CR55] Pingault V, Ente D, Dastot-Le Moal F, Goossens M, Marlin S, Bondurand N (2010). Review and update of mutations causing Waardenburg syndrome. Hum Mutat.

[CR56] Pinz H, Pyle LC, Li D (2018). De novo variants in myelin regulatory factor (MYRF) as candidates of a new syndrome of cardiac and urogenital anomalies. Am J Med Genet A.

[CR57] Said MB, Chouchene E, Salem SB (2013). Posterior microphthalmia and nanophthalmia in Tunisia caused by a founder c.1059_1066insC mutation of the PRSS56 gene. Gene.

[CR58] Schaffer AA, Gupta SK, Shriram K, Cottingham RW (1994). Avoiding recomputation in linkage analysis. Hum Hered.

[CR59] Shen L, Melles RB, Metlapally R, Barcellos L, Schaefer C, Risch N, Herrinton LJ, Wildsoet C, Jorgenson E (2016). The association of refractive error with glaucoma in a multiethnic population. Ophthalmology.

[CR60] Simpson CL, Wojciechowski R, Oexle K (2014). Genome-wide meta-analysis of myopia and hyperopia provides evidence for replication of 11 loci. PLoS One.

[CR61] Spitznas M, Gerke E, Bateman JB (1983). Hereditary posterior microphthalmos with papillomacular fold and high hyperopia. Arch Ophthalmol.

[CR62] Stohr H, Marquardt A, White K, Weber BH (2000). cDNA cloning and genomic structure of a novel gene (C11orf9) localized to chromosome 11q12→q13.1 which encodes a highly conserved, potential membrane-associated protein. Cytogenet Cell Genet.

[CR63] Sun W, Huang L, Xu Y, Xiao X, Li S, Jia X, Gao B, Wang P, Guo X, Zhang Q (2015). Exome sequencing on 298 probands with early-onset high myopia: approximately one-fourth show potential pathogenic mutations in RetNet genes. Invest Ophthalmol Vis Sci.

[CR64] Sun X, Dai Y, Chen Y, Yu DY, Cringle SJ, Chen J, Kong X, Wang X, Jiang C (2017). Primary angle closure glaucoma: what we know and what we don’t know. Prog Retin Eye Res.

[CR65] Sundin OH, Leppert GS, Silva ED (2005). Extreme hyperopia is the result of null mutations in MFRP, which encodes a Frizzled-related protein. Proc Natl Acad Sci USA.

[CR66] Tarabishy AB, Alexandrou TJ, Traboulsi EI (2007). Syndrome of myelinated retinal nerve fibers, myopia, and amblyopia: a review. Surv Ophthalmol.

[CR67] Vardarajan BN, Barral S, Jaworski J (2018). Whole genome sequencing of Caribbean Hispanic families with late-onset Alzheimer’s disease. Ann Clin Transl Neurol.

[CR68] Vingolo EM, Steindl K, Forte R, Zompatori L, Iannaccone A, Sciarra A, Del Porto G, Pannarale MR (1994). Autosomal dominant simple microphthalmos. J Med Genet.

[CR69] Wang P, Yang Z, Li S, Xiao X, Guo X, Zhang Q (2009). Evaluation of MFRP as a candidate gene for high hyperopia. Mol Vis.

[CR70] Wasmann RA, Wassink-Ruiter JS, Sundin OH, Morales E, Verheij JB, Pott JW (2014). Novel membrane frizzled-related protein gene mutation as cause of posterior microphthalmia resulting in high hyperopia with macular folds. Acta Ophthalmol.

[CR71] Wojciechowski R, Congdon N, Bowie H, Munoz B, Gilbert D, West S (2005). Familial aggregation of hyperopia in an elderly population of siblings in Salisbury, Maryland. Ophthalmology.

[CR72] Xiao X, Li S, Jia X, Guo X, Zhang Q (2016). X-linked heterozygous mutations in ARR3 cause female-limited early onset high myopia. Mol Vis.

[CR73] Xu Y, Guan L, Xiao X (2016). Identification of MFRP mutations in Chinese families with high hyperopia. Optom Vis Sci.

[CR74] Yalcindag FN, Atilla H, Batioglu F (2011). Optical coherence tomography findings of retinal folds in nanophthalmos. Case Rep Ophthalmol Med.

[CR75] Zhang Q, Zulfiqar F, Xiao X, Riazuddin SA, Ayyagari R, Sabar F, Caruso R, Sieving PA, Riazuddin S, Hejtmancik JF (2005). Severe autosomal recessive retinitis pigmentosa maps to chromosome 1p13.3-p21.2 between D1S2896 and D1S457 but outside ABCA4. Hum Genet.

