# Supplementary material for: Novel truncation mutations in MYRF cause autosomal dominant high hyperopia mapped to 11p12–q13.3
Source: Hum Genet. 2019 Jun 6;138(10):1077–90. doi: 10.1007/s00439-019-02039-z (PMC6745028; doi:10.1007/s00439-019-02039-z)
Supplement: Supplementary file 1 — Supplementary material 1 (DOCX 19 kb) [file 439_2019_2039_MOESM1_ESM.docx]

Journal: Human Genetics

Novel truncation mutations in *MYRF* cause autosomal dominant high hyperopia mapped to 11p12-q13.3

Xueshan Xiao,^1#^ Wenmin Sun,^1#^ Jiamin Ouyang,^1^ Shiqiang Li,^1^ Xiaoyun Jia,^1^ Zhiqun Tan,^2^ J. Fielding Hejtmancik,^3^ Qingjiong Zhang^1*^

^1^State Key Laboratory of Ophthalmology, Zhongshan Ophthalmic Center, Sun Yat-sen University, 54 Xianlie Road, Guangzhou 510060, China. ^2^Institute for Memory Impairments and Neurological Disorders, University of California, Irvine, California, USA. ^3^Ophthalmic Genetics and Visual Function Branch, National Eye Institute, National Institutes of Health, Bethesda, Maryland, USA.

^#^These authors contributed equally to this work.

*Corresponding author: Qingjiong Zhang, MD, PhD, Pediatric and Genetic Eye Clinic, Zhongshan Ophthalmic Center, Sun Yat-sen University, 54 Xianlie Road, Guangzhou 510060, China. Phone: (+86)-20-66677083;

Email: [zhangqji@mail.sysu.edu.cn](mailto:zhangqji@mail.sysu.edu.cn) or [zhangqingjiong@gzzoc.com](mailto:zhangqingjiong@gzzoc.com)

| Supplementary Table 1. Sequences of primers and MOs used in this study | |  |
| --- | --- | --- |
| Primer name | Primer sequence (5'-3') | Product length |
| *MYRF* Sanger-exon24-F | TCCCCCATAAGGAAGGGTAG |  |
| *MYRF* Sanger-exon24-R | TATCCCACCATGCAGGAAAT | 351bp |
| *MYRF* Sanger-exon25-F | GCCCTGGTTATTATGCGGTG |  |
| *MYRF* Sanger-exon25-R | AGCTCACCATCCAGTTCTCC | 697bp |
| *MYRF* Sanger-exon26-27-F | TCCTTCATAAAACTCCCCACA |  |
| *MYRF* Sanger-exon26-27-R | TTTCACCAGTCTCGGGTAGG | 590bp |
| *myrf* MO efficiency test-F | GCATGAATTCACCCGCAGATATGTACTGCGAAG |  |
| *myrf* MO efficiency test-R | GTTAGGATCCGTTCCTGGTGCGTACTGTCC | 709bp |
| *myrf* MO rescue-F | GCTAGAATTCACCATGGAGACGGTGATAGATGA |  |
| *myrf* MO rescue-R | ATCGTCTAGATTAGCATGTGCTGTGAATGA | 3104bp |
| *myrf* MO | CAATCACGGTTTCCATTCTAGCGGC |  |
| std MO | CCTCTTACCTCAGTTACAATTTATA |  |
| Note: Sanger means mutation validation in three families by Sanger sequencing. F=forward; R=reverse. | | |

| Supplementary Table 2. The six truncation variants in MYRF in the current study and previous studies. | | | | | | | | |  |
| --- | --- | --- | --- | --- | --- | --- | --- | --- | --- |
| Exon/Intron | Nucleotide change | Amino acid change | Status | Allele frequency in | | | | Disease | Reference |
|  |  |  |  | Cases | 1000G | gnomAD_all | gnomAD_EAS |  |  |
| Exon8 | c.1254_1255dup | p.Thr419Argfs*14 | Het | 1/1 | None | None | None | Cardiac and urogenital anomalies | Chitayat et al., 2018 |
| IVS17 | c.2336+1G>A | / | Het | 1/2 | None | None | None | Cardiac and urogenital anomalies | Pinz et al., 2018 |
| Exon20 | c.2518C>T | p.Arg840* | Het | 1/2 | None | None | None | Cardiac and urogenital anomalies | Pinz et al., 2018 |
| IVS24 | c.3194+2T>C | / | Het | 1/122 | None | None | None | High hyperopia | This study |
| Exon25 | c.3274_3275del | p. Leu1093Profs*22 | Het | 1/122 | None | None | None | High hyperopia | This study |
| Exon27 | c.3377del | p.Gly1126Valfs*31 | Het | 1/122 | None | None | None | High hyperopia | This study |
